# Impacts of climate and reduced ice cover on trophic structure and community dynamics in a high latitude lake

**DOI:** 10.1007/s00442-026-05957-8

**Published:** 2026-08-14

**Authors:** Francesca Pilotto, Knut Marius Myrvold, Randi Saksgård, Knut Andreas Eikland, Birger Skjelbred, Stefano Larsen, Gunnar Halvorsen, Trygve Hesthagen, Thomas C. Jensen

**Affiliations:** 1 Norwegian Institute for Nature Research (NINA), Oslo, Norway; 2 Norwegian Institute for Nature Research (NINA), Lillehammer, Norway; 3 Norwegian Institute for Nature Research (NINA), Trondheim, Norway; 4 Norwegian Institute for Water Research (NIVA), Oslo, Norway; 5 Research and Innovation Centre, Edmund Mach Foundation, San Michele all’Adige, Italy; 6 NBFC- National Biodiversity Future Center, Palermo, Italy

**Keywords:** Phytoplankton, Zooplankton, Fish, Ice phenology, Community stability

## Abstract

Climate-driven environmental change can alter both the structural and dynamic properties of ecological communities. With ongoing warming, high-latitude lakes experience reduced ice cover, higher growing-season temperatures, and altered hydrology, potentially triggering cascading effects across lake ecosystems. We investigated long-term variation (1994–2022) in ice phenology, temperature, magnitude of spring floods, and pelagic communities in an oligotrophic subalpine lake in Norway. We analysed the structure and dynamics of phytoplankton, zooplankton, and fish communities, as well as synchrony across trophic groups. Long-term records indicated declining ice-cover duration and increasing growing-season temperatures, although only the reduction in ice-cover duration was significant within the 1994–2022 study period. Phytoplankton richness and biovolume increased, zooplankton communities increased in diversity but declined in temporal stability, and fish community structure changed, with brown trout becoming more abundant at the expense of arctic char. We also observed shifts in synchrony between trophic groups, suggesting changes not only in community organisation but potentially in interspecific interactions. Given the observational, correlative nature of the study, causal attribution remains limited. Although some biological metrics showed significant associations with climate-related variables, several metrics that changed over time were not related to the measured abiotic drivers, indicating partial coupling between environmental variability and community structure and dynamics. This limited coupling may reflect the relatively modest magnitude of environmental change that occurred over the study period. Overall, our results document cross-trophic restructuring of pelagic communities in a high-latitude oligotrophic lake and underscore the importance of long-term monitoring for documenting and anticipating future shifts.

**Supplementary Information:**

The online version contains supplementary material available at https://doi.org/10.1007/s00442-026-05957-8.

## Introduction

Climate-driven changes in environmental conditions, such as warming, shifts in ice phenology, and hydrological variability, have the potential to alter the structure and dynamics of ecological communities (Ives and Carpenter 2007). Environmental changes can alter structural properties, including species richness, diversity, and relative abundances, by shifting environmental filters and favouring species suited to novel conditions, while disfavouring others. This process reshapes community composition, biotic interactions, and resource partitioning (Tilman 1999). At the same time, environmental variability influences community dynamics by altering the magnitude and synchrony of population fluctuations, with consequences for the stability of ecosystem functioning (Ives and Carpenter 2007). Ecological theory suggests that these dimensions are interconnected: structural diversity can influence temporal dynamics, while interactions and covariances among populations modulate stability and synchrony (Tilman 1999; Thibaut and Connolly 2013). However, considering both structural and dynamical community responses to environmental drivers remains uncommon, yet such an approach can provide valuable insight into changes in ecosystem stability and functioning under climate warming.

Climate warming has pervasive effects on lake ecosystems worldwide, with ice-covered lakes warming more rapidly than other lakes (O’Reilly et al. 2015). Increasing temperatures cause changes in lake ice phenology, including later ice-on and earlier ice-off dates, resulting in shorter periods of ice cover. In mountainous and cold-climate regions, where snow and ice are integral components of the hydrological cycle, climate warming also reshapes the timing and magnitude of runoff as the cryosphere declines (IPCC 2019). In Norway, projected increases in temperature and changes in seasonal precipitation are expected to shift snowmelt dynamics and alter streamflow patterns (Skålevåg and Vormoor 2021), with consequences for lake water balance, residence time, and nutrient inputs. These shifts in ice phenology, hydrological regimes, and water temperature can have cascading effects on lake ecosystems, influencing evaporation and water budgets, mixing regimes, biogeochemical cycling, algal blooms and biotic assemblages (Paerl and Huisman 2008; Wang et al. 2018; Woolway et al. 2020; Hebert et al. 2021), with potential impacts on lake functioning and ecosystem services, including drinking water and recreational use. For example, toxic algal blooms in the oligotrophic Dickson Lake (Ontario, Canada) have been linked to anomalous ice phenology, combined with elevated temperatures and low wind speeds, likely reducing spring mixing and creating favourable conditions for cyanobacteria growth (Favot et al. 2019).

Lake ice phenology directly affects the availability of light and nutrients in the water column (Weyhenmeyer 2009; De Senerpont Domis et al. 2013). Changes to the duration of the ice-covered period may in turn alter the composition, biomass and succession of lake biota during winter and the following ice-free season (Hampton et al. 2017). For example, delayed ice-on has been observed to increase algae densities in early winter, raise cyclopoid copepod abundance during the ice-cover period, and alter the zooplankton’s fatty acid composition, indicating enhanced winter survival and increased energy storage (Hebert et al. 2021). The number and magnitude of phytoplankton peaks have been shown to abruptly increase with shortening ice cover duration in oligotrophic lakes in Sweden (Weyhenmeyer et al. 2013). Similarly, shorter ice cover durations have been linked to earlier and more pronounced spring phytoplankton peaks, particularly for diatoms, in shallow lakes (Adrian et al. 1999).

While previous studies suggest a boosting effect of shorter ice cover on phytoplankton production (Adrian et al. 1999; Weyhenmeyer et al. 2013; Hebert et al. 2021), the effects of ice phenology and warming on zooplankton and fish appear more variable across geographic regions, taxonomic groups, and community compositions. A study of Alaskan lakes (59 °N) showed that zooplankton exhibited species-specific responses to early spring ice break-up and warming, without a boost in total zooplankton productivity (Carter and Schindler 2012). In a sub-alpine Californian lake (41 °N), reduced ice cover and increased water temperature were associated with decreased pelagic zooplankton production, increased littoral zoobenthos, and reduced body condition of brook trout (*Salvelinus fontinalis*) (Caldwell et al. 2020). The negative impact on the fitness of brook trout was due to their avoidance of the relatively warmer littoral zone, limiting their access to zoobenthos and forcing them to rely on less nutritious pelagic zooplankton (Caldwell et al. 2020). On the other hand, the annual growth of young-of-the-year arctic char (*S. alpinus*) appeared faster in years with warmer summers and milder winters with earlier ice break-up in high-arctic lakes in Svalbard (78–79 °N) exhibiting overall temperatures well below the thermal optima for salmonids (Svenning et al. 2024). Ice phenology can also influence fish species interactions, as shown for Norwegian lakes (59–70 °N), where increased ice cover reduced brown trout (*Salmo trutta*) biomass, but only when arctic char were also present (Helland et al. 2011).

Community stability, defined as the temporal variability in the relative abundance or biomass of the constituent species, determines the community’s ability to maintain its functions under environmental changes. According to the portfolio effect (Thibaut and Connolly 2013), stability tends to be higher in more diverse communities with a high degree of population asynchrony. This occurs because individual populations can display compensatory dynamics as they fluctuate out of phase with one another (i.e. asynchronously; O’Connor et al. 2023). However, environmental changes can impact stability. Previous studies have shown that warming temperatures can disrupt community stability by increasing population synchrony (Larsen et al. 2024). Warming is also expected to cause phenological mismatches between resource availability and consumer needs (Thackeray et al. 2010), a process referred to as trophic asynchrony. Despite these expectations, the effects of warming on community dynamics and asynchrony across trophic levels in lakes have only occasionally been studied (Winder and Schindler 2004). Recent findings demonstrated that warming reduced the stability within phytoplankton and zooplankton communities in a subtropical lake and led to greater synchrony in the annual population fluctuations of phytoplankton producers and zooplankton consumers (Rao et al. 2024). This was likely due to the extended growing seasons, which allowed both phytoplankton and zooplankton to thrive for longer periods, leading to greater overlap in their annual population cycles (Rao et al. 2024). On the other hand, a modelling study across European lakes predicted that warming could cause asynchrony between earlier phytoplankton blooms and peak consumer (*Daphnia*) abundance in lakes where blooms are regulated by ice cover (Gronchi et al. 2023).

Previous studies suggest that warming, changing ice phenology and hydrology may have a pervasive effect on lake biota; however, how those effects propagate across the different trophic levels of the food web in ice-covered lakes remains unclear. This uncertainty persists because such investigations require long-term data, and the effects of climate-related variables may be influenced by factors such as the magnitude of ice cover reduction and lake-specific characteristics. Additionally, climate effects could be masked by the impacts of anthropogenic pressures, such as land use and eutrophication.

**Fig. 1:**
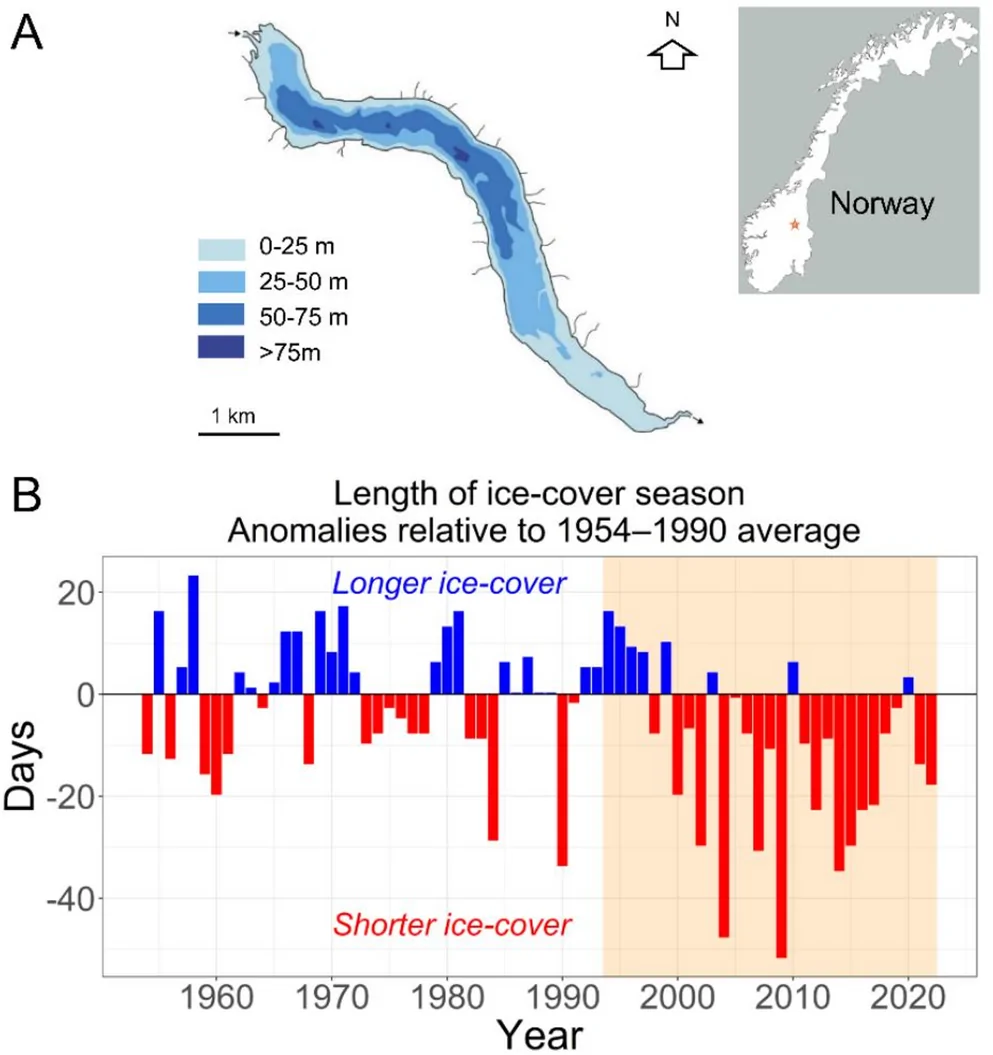
Bathymetric map of lake Atnsjøen in Norway (**A**) and annual ice cover duration, shown as deviations (number of days) from the average ice cover length from 1954–1990 (**B**). Red bars indicate shorter-than-average ice cover duration and blue bars indicate longer duration. The study period (1994–2022) is highlighted

In this study, we analysed time-series of phytoplankton, zooplankton and fish communities from 1994 to 2022, in an oligotrophic lake in a semi-pristine catchment in Norway (62 °N, Fig. 1A). We investigated how the structure (i.e. abundance, richness, taxonomic composition, diversity) and dynamics (community stability, as well as population and trophic asynchrony) of lake biota changed over time, and whether those changes were associated with variations in ice phenology, temperature and magnitude of spring floods. By examining long-term dynamics across multiple trophic levels in a minimally impacted lake, this study offers a unique opportunity to appraise the impact of changing climate-related variables on lake biota. We anticipate that the length of the ice-cover period has been shrinking over the past decades, in accordance with national reports that showed an acceleration in the trends of earlier ice-off and later ice-on since the early 1900s (L’Abée-Lund et al. 2021). We asked the following overarching research questions: (Q1) How have the structure and dynamics of communities of phytoplankton, zooplankton and fish changed in lake Atnsjøen in the past three decades? (Q2) Were those community changes correlated with changes in climate and ice phenology?

We expected that a shortening of the ice cover duration, increasing temperatures in the growing season and altered magnitude of spring floods were associated with increasing phytoplankton biovolume and with shifts in phytoplankton composition, following Weyhenmeyer et al. (2013), who found higher richness and biomass of diatoms, chlorophytes, and cyanobacteria under shorter ice-cover periods. We also expected that such changes in climate and phytoplankton assemblages would affect higher trophic levels, leading to shifts in composition and increased densities of zooplankton (particularly cladocerans and calanoid copepods; Carter and Schindler 2012) and fish consumers (favouring brown trout over arctic char; Helland et al. 2011). Finally, we expected that higher temperatures and shorter ice cover would lead to more synchronous populations within trophic levels, resulting in more unstable communities (Larsen et al. 2024). We also expected reduced synchrony between trophic levels due to mismatches between resource availability and consumer demand (Thackeray et al. 2010).

## Materials and methods

### Study area

Lake Atnsjøen (61° 52’51 N, 10° 09’55 E) is an oligotrophic dimictic lake with a surface area of 4.8 km^2^ and catchment surface area of 457 km^2^. The maximum and mean depths are 80 m and 35.4 m. The retention time of the lake is about 6 months (Halvorsen 2004). The highest annual discharge typically occurs in spring during snowmelt, usually between May and June, but rain-driven floods can occur in summer and fall. The lake is situated at 701 m above sea level, in an area with continental climate. A large portion of the catchment area (ca. 85%) is situated above the tree line (approx. 1000 m a.s.l.), the remaining part is mostly forested with pine (*Pinus sylvestris*) and birch (*Betula pubescens*) as the dominating trees (Tvede and Halvorsen 2004). Although there are significant deposits of quaternary and fluvial rocks in some places, the catchment is primarily composed of feldspar quartzite. There are no glaciers in the catchment, but during cold summers, patches of snow remain at high elevations and affect the temperature of the lake. The lake is typically ice covered from late November to late May, and snow cover typically lasts from November to early May. In summer, the lake is weakly stratified with a thermocline usually situated at approximately 10 m depth (Halvorsen 2004).

The catchment of Lake Atnsjøen is relatively unaffected by human activities due to its remote location and the fact that large part of it lies within the Rondane National Park. The population in the area peaked in the first half of the 20th century and has declined since around 1950 (Jensen et al. 2020). Even at its highest (estimated as 0.46 inhabitants/km²), population density was low by Norwegian, European, and North American standards, highlighting minimal human pressure on the lake (Jensen et al. 2020). Traditionally, fishing in Lake Atnsjøen was an important supplementary food source for local residents (Brænd 1989, 2007, 2009), but its significance has diminished with the declining human presence in the area. Today, there is still recreational fishing in the lake, but it is of minor scale compared to traditional fishing, and overall fishing pressure has decreased since the 1980s (H.E. Nesset, pers. comm.). This trend of declined fishing pressure aligns with paleolimnological evidence showing an increase in large cladocerans, i.e. a proxy for reduced fish predation (Jeppesen et al. 1996), between 1980 and 2012 (Jensen et al. 2020).

## Biotic data

### Phytoplankton and zooplankton

We collected samples of phytoplankton and zooplankton each year, one time per month from June to October, from 1990 to 2022 (sampling location is shown in Supplementary Fig. 1). We used data from 1994 to 2022 to conform with the span of available fish data.

We collected phytoplankton from integrated samples from the top 10 m of the water column and fixed the samples with Lugol’s solution. Details on procedures for sampling and counting of phytoplankton, following the standards EN 15204:2006 and EN 16695:2015, are described by Brettum and Halvorsen (2004). In this work, we grouped taxa into phyla or classes to minimize the impact of changed taxonomic expertise throughout the time series. Because the temporal resolution was insufficient to robustly characterise intra-annual dynamics, we focused our analyses on the interannual variation using yearly means. We calculated total and phylum- or class-specific yearly values as the mean biovolume across the five sampling occasions (1 sample per month from June to October) within each year.

Procedures for sampling and counting of zooplankton are described in detail by Halvorsen et al. (2004). Briefly, we collected the samples using a Schindler sampler at 0, 1, 2, 4, 6, 8, 10, 15, 20, 30 and 50 m depth, with five replicates per depth stratum. We filtered these samples through a 45 μm mesh and preserved with Lugol’s solution. We ran the analyses at the finest possible taxonomic level, in most cases at the species level. We calculated zooplankton density (ind. l^− 1^) from those quantitative samples and adjusted for the relative contribution of the volume of each depth layer of the lake, taking into account the bathymetry of the lake. As for phytoplankton, we calculated yearly mean total density and mean species-specific density as the average across the five sampling dates for each year. We included copepod nauplii only in the total density estimates but we excluded them from the taxon-specific and diversity analyses because we did not distinguish between cyclopoid and calanoid nauplii in the quantification of the samples.

## Fish

The fish community in lake Atnsjøen consists of four species (Hesthagen and Sandlund 2004): brown trout, arctic char, common minnow (*Phoxinus phoxinus*) and alpine bullhead (*Cottus poecilopus*). Brown trout and arctic char are the only fish species occurring in the pelagic zone of the lake. In contrast, common minnow is primarily benthic, while alpine bullhead is strictly benthic; in Lake Atnsjøen, both species are confined to the littoral zone and do not occur in pelagic habitats.

We conducted test fishing with pelagic (floating) and benthic gillnets in August from 1985. The nets have varied in numbers and type over the years. To ensure consistency in the data, we only used data from 1994 onwards, as the net type changed from Jensen nets to Nordic nets between 1993 and 1994. The number of pelagic nets varied among sampling years between 2 (total surface area of net: 648 m^2^) and 6 (total net surface: 1944 m^2^), and were deployed across three stations over the study period (Supplementary Fig. 1). The benthic nets (Nordic nets) were deployed consistently at eight stations (total surface area: 2025 m^2^; Supplementary Fig. 1). We computed the catch per unit effort (CPUE) for the benthic and for the pelagic nets separately, as the total number of individuals divided by the total surface area of the nets of the two types. We then computed the yearly (total) CPUE as the average of CPUE (ind. m^− 2^) for the benthic and pelagic zone for each species (Supplementary Fig. 2).

## Abiotic data

### Climate data

We obtained ice-cover data from Iskart (http://www.iskart.no). Those data included the date of ice break up and the date when the lake freezes up for each year since 1954. For each sampling year, we then computed the length of the ice-cover period of the previous winter, which decreased from an average of 192 days in the first 5 years of the study (1994–1998) to 176 days in the last five years (2018–2022).

We obtained data on daily air temperature and lake outlet discharge from the Norwegian Water Resources and Energy Directorate. Specifically, we downloaded daily air temperatures at the lake from the 1 × 1 km grid time series data for Norway (https://api.nve.no/doc/gridtimeseries-data-gts; available from 1957) and daily lake outlet discharges from the hydrological database Sildre (https://sildre.nve.no; data available from September 1984).

We computed the mean temperature of the growing season for each year as the mean temperature between the date of ice-off and the date of the biological sampling in September. For each year, we also computed average spring discharge (1st March to 30th June), as a measure of the magnitude of the spring flood.

### Data analysis

#### Biotic metrics

Based on yearly taxon averages, we computed a series of metrics describing the structure and dynamics of each biological community for each year. Here we use the term “community” to indicate the assemblage of each biotic group: phytoplankton, zooplankton and fish communities. We computed a metric of total abundance (i.e. total biovolume for phytoplankton, density for zooplankton and CPUE for fish), taxonomic richness, and Shannon diversity (hereafter “diversity”) of each community. We also conducted Principal Coordinates Analysis (PCoA) for each community separately, using the first axis of each PCoA as a metric to describe community composition (hereafter referred to as “composition”). We performed the PCoA analyses using the *cmdscale* function in the “stats” package of base R (R Core Team 2019), applied to Euclidean distance matrices derived from Hellinger-transformed data. Hellinger transformation reduces the influence of dominant taxa and ensures the suitability of Euclidean distance in community analyses (Legendre and Gallagher 2001). The first PCoA axes represented the 36.8%, 36.2% and 78.0% of the variance of phytoplankton, zooplankton and fish communities, respectively (Supplementary Fig. 3).

Following the equations reported by Rao et al. (2024), we computed community stability and population asynchrony for each community (phytoplankton, zooplankton and fish) separately, and we computed trophic asynchrony between resources and consumers. We computed these metrics in sliding windows of 5 years. To reduce redundancy from overlapping years in the 5-year sliding windows, we included only values from every second sliding window in further analyses. Community stability is the temporal stability of the total abundance of a community and was computed as:

$$\:Community\:stability=\frac{{\mu\:}_{T}}{{\sigma\:}_{T}}$$

where $\:{\mu\:}_{T}$ is the mean and $\:{\sigma\:}_{T}\:$ is the standard deviation of the total abundance.

Population asynchrony is the temporal inconsistency of the variances of the populations within a community and was computed as:

$$\:Population\:asynchrony=1-\frac{{{\sigma}_{T}}^{2}}{{\left(\sum_{i=1}^{N}{\sigma}_{i}\right)}^{2}}$$

where * N* is the number of populations in the community and $\:{\sigma\:}_{i}\:$ is the standard deviation of the population * i*. High values of population asynchrony mean that the populations within the community do not fluctuate in the same way or at the same time, for example when some species respond positively to a change, while others respond negatively or not at all.

Finally, trophic asynchrony measures the temporal mismatch between the abundances of resources and consumers (Rao et al. 2024). We computed it as:

$$\:Trophic\:asynchrony=1-\frac{{{\sigma\:}_{(r+c)}}^{2}}{{({\sigma\:}_{r}+{\sigma\:}_{c})}^{2}}$$

where $\:{\sigma\:}_{\left(r+c\right)}\:$is the standard deviation of the total abundance of resources and consumers together, and $\:{\sigma\:}_{\left(r\right)}\:$ and $\:{\sigma\:}_{\left(c\right)}$ are the standard deviations of the abundance of resources and consumers, respectively. Differently to Rao et al. (2024), who computed trophic asynchrony between total phytoplankton biomass and total zooplankton biomass, we focused on specific trophic relationships by computing trophic asynchrony for (1) total phytoplankton biovolume and density of zooplankton herbivores, (2) density of Cladocera and arctic char CPUE, (3) density of Cladocera and brown trout CPUE. This approach reflects the fact that cladocerans are the predominant zooplanktonic food resource for the two pelagic species (brown trout and arctic char) in this lake during the growing seasons (Saksgård and Hesthagen 2004), although they also consume benthic invertebrates (which are not studied here). As trophic asynchrony is based on the variance of abundances and is thus affected by different measurement, we scaled the biovolume or density or CPUE of the trophic levels by min-max as (x – x_min_) / (x_max_ – x_min_). Zooplankton species were classified as herbivores following Merz et al. (2023).

### Trend and correlation analysis

To answer Q1, we analysed the trends of biological metrics during the studied period. For that, we fitted generalized additive models (GAM) with each metric as response variable and year included as smooth term (k = 5). Models were fitted using restricted maximum likelihood (REML). We ran similar GAM models also for analysing the trends of climatic variables, both for the study period (1994–2022) and for the entire available abiotic time series.

To identify the correlations between biotic metrics and climatic variables (Q2), we used GAM, with biotic metrics as response variables and climatic variables as explanatory variables (smoothers; k = 4). For climatic variables, we included the day of the year corresponding to the date when the lake froze up the autumn before the biological sampling (hereafter “ice-on”), the day of the year corresponding to the date of ice break up in the year of the biological sampling (hereafter “ice-off”), the mean temperature in the growing season and the spring discharge. The length of the ice-cover period was correlated with ice-off (Pearson *r* = 0.71) and with ice-on (Pearson *r* = -0.88). We therefore kept ice-on and ice-off and excluded length of the ice-cover period from the models. The correlation coefficients between pairs of the remaining climatic variables were below 0.4. We did not apply GAM models to fish community richness because of its very limited interannual variation (3–4 species). For stability and asynchrony metrics we used as explanatory variables the average values of the climatic variables within the 5-year sliding windows that were used for computing the biotic metrics.

We fitted GAMs models using the function *gam* in the R package “mgcv” (version 1.9-1; Wood 2011). We used a Gaussian distribution family for all models, except those modelling phytoplankton and zooplankton group biovolumes or densities, which employed a negative binomial distribution, and fish species CPUE, which was modelled using a quasi-Poisson distribution. We conducted model diagnostics by examining the residual distribution using the *appraise* function from the R package “gratia” version 0.9.0 (Simpson and Singmann 2022) and performed additional diagnostic checks using the *gam.check* function from the R package “mgcv” (Wood 2011).

## Results

Long-term abiotic records (1954–2022) showed a decline in ice-cover duration (*p* = 0.004), driven primarily by earlier ice-off dates (*p* < 0.001), as well as increasing growing-season temperatures (*p* < 0.001; Supplementary Fig. 4). From 2000 onwards, ice-cover duration remained consistently shorter than the historical average (1954–1990; Fig. 1B).

When limiting the analysis to the period with available biotic data (1994–2022), the average duration of ice cover declined from 192 days in the first five years to 176 days in the last five years (*p* = 0.015). However, no significant trends were observed for either the ice-on or ice-off dates (*p* > 0.05), although they shifted on average by 6 days (from Julian day 328 to 322) and 10 days (from Julian day 149 to 139), respectively. The mean temperature in the growing season and the magnitude of the spring flood did not show significant trends during 1994–2022 (*p* > 0.05), although considerable spring floods were registered in 1995, 2008 and 2013 (Supplementary Fig. 4).

**Fig. 2:**
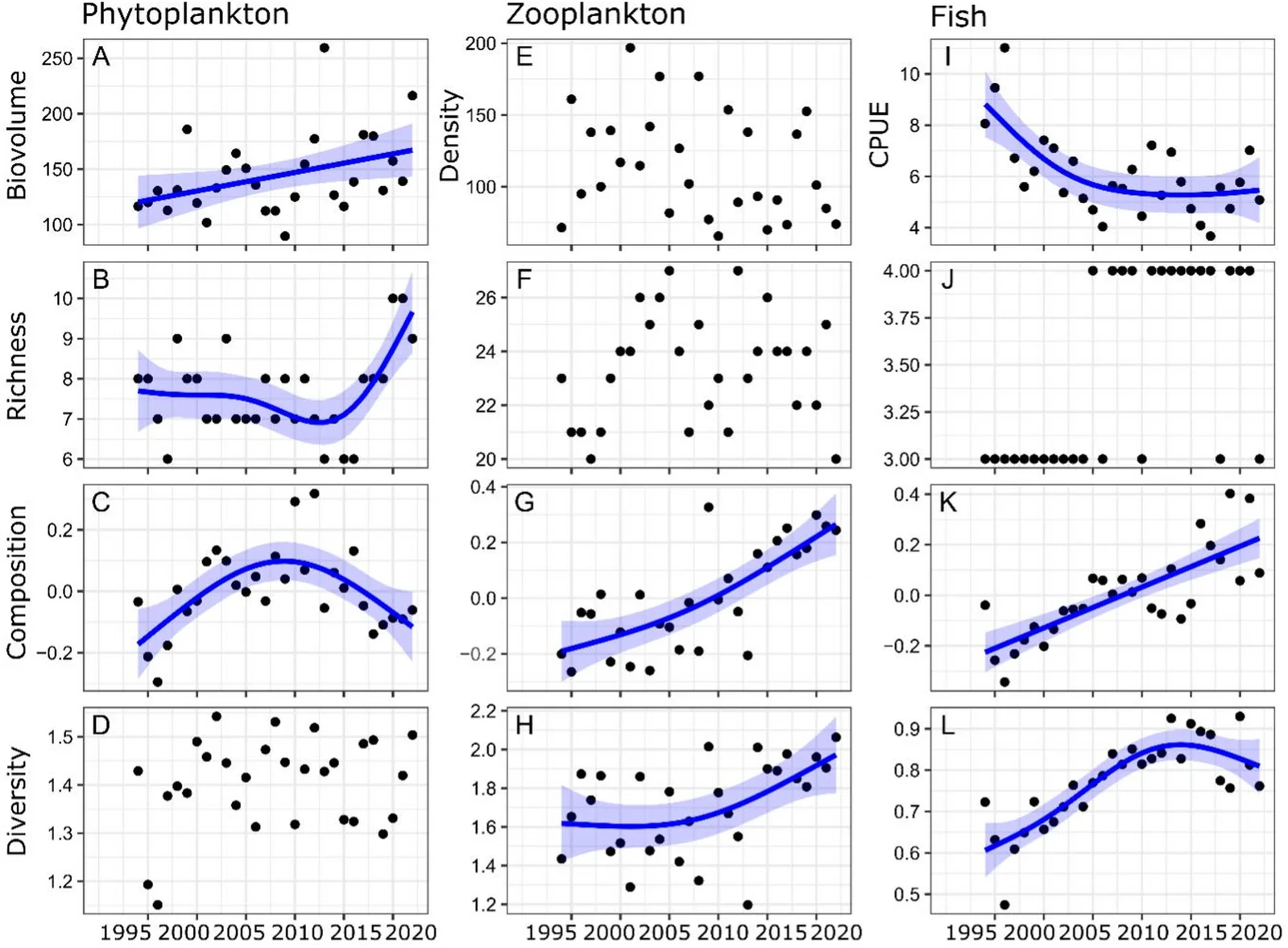
Changes in the structure of phytoplankton (**A-D**), zooplankton (**E-H**) and fish (**I-L**) communities through time (*N* = 29 annual observations, 1994–2022). Phytoplankton biovolume is expressed as mm³ m⁻³, zooplankton density as ind. L⁻¹, and fish CPUE as number of individuals. Points show observed values. Blue lines and ribbons show the fitted GAM smooth and 95% C.I., only significant smooths are shown

The biotic communities of lake Atnsjøen changed substantially during the study period. For phytoplankton, total biovolume steadily increased through time (Fig. 2A), richness increased towards the end of the time series (ca. after 2015; Fig. 2B), whereas changes in community composition showed a unimodal curve with maximum at ca. 2010 (Fig. 2C). Those changes reflected an increase in the biovolume of Charophyta/Chlorophyta and Haptophyta and a decline of Dinophyceae thorough time, a relatively lower biovolume of the dominating group Cryptophyta around 2005, and a unimodal change in Picoplankton biovolume with maximum at around 2010 (Supplementary Fig. 5). Also noteworthy was an increase in biovolume and incidence of cyanobacteria in the most recent years. While this group was mostly absent until 2018, its biovolume and presence across sampling dates increased from 2019 onward (Supplementary Figs. 5–6). Phytoplankton diversity showed no trend (Fig. 2D).

Zooplankton total density did not change through time nor did richness. However, zooplankton community composition changed steadily and the diversity increased, especially towards the end of the time series (Fig. 2E - H). These changes mirrored an increase in the density of copepods (both Cyclopoida and Calanoida) and a decline in the dominance of rotifers (Supplementary Fig. 5).

Fish CPUE declined through time, community composition changed directionally, and diversity increased up to ca. year 2015 (Fig. 2I - L). Overall, these changes reflected a shift from a dominance of arctic char at the beginning of the time series to a more equal representation of char and brown trout in the central part of the time series, to a dominance of brown trout towards the end of the time series (Supplementary Fig. 5). Common minnow and alpine bullhead represented only a small fraction of the total catch.

**Fig. 3:**
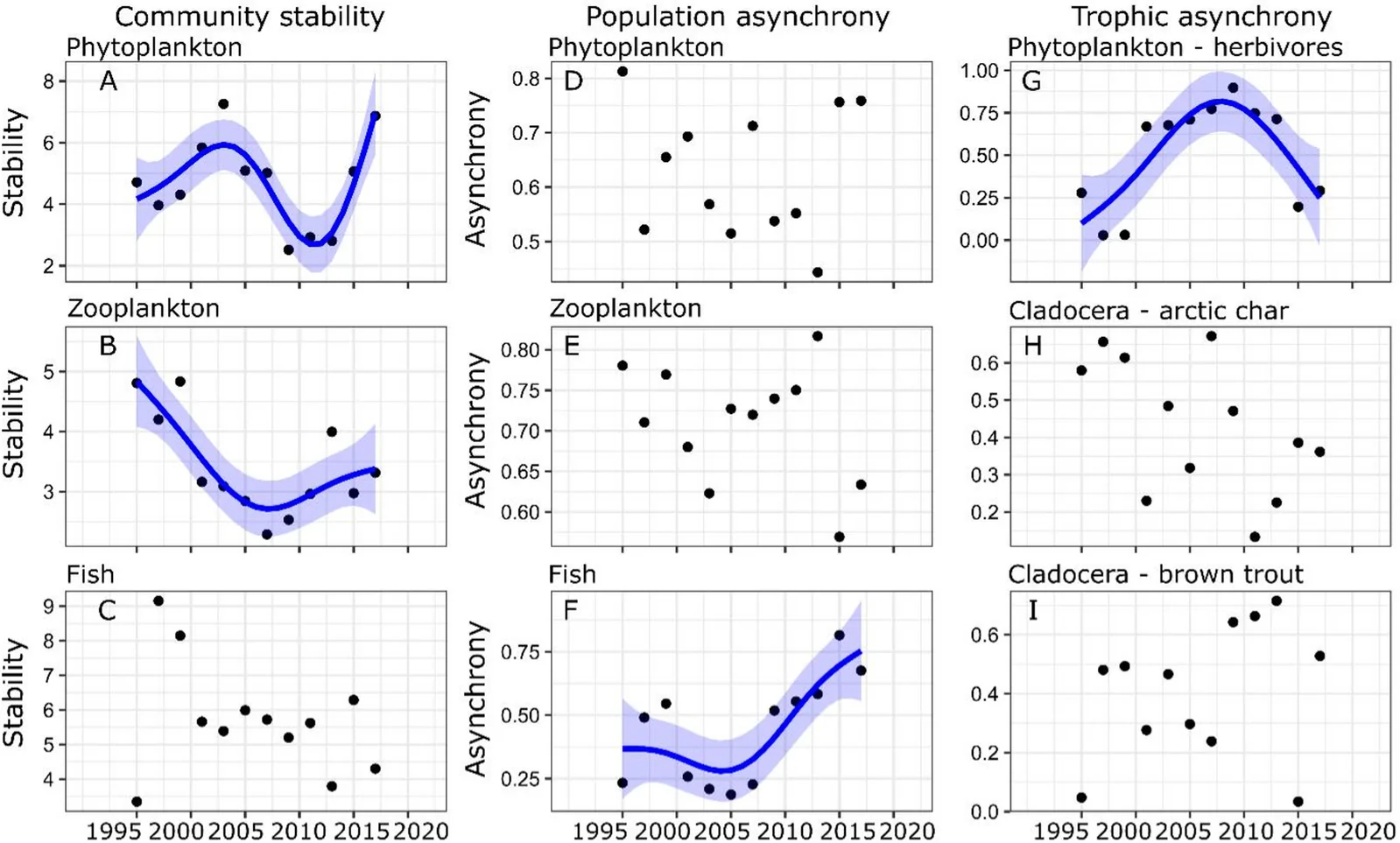
Changes in the dynamics of the biotic communities at Lake Atnsjøen through time, as shown by community stability (**A-C**) and population asynchrony (**D-F**) of phytoplankton, zooplankton and fish, and trophic asynchrony (**G-I**) between (**G**) phytoplankton and zooplankton herbivores, (**H**) Cladocera and arctic char, and (**I**) Cladocera and brown trout. Points represent moving-window values assigned to the first year of each window (*N* = 12); blue lines and ribbons show the fitted GAM smooth and 95% C.I., only significant smooths are shown

While the phytoplankton community showed non-linear changes in stability and no changes in population asynchrony through time (Fig. 3A, D), the zooplankton community became less stable from the beginning of the time series up to ca. 2010 when stability levelled off (Fig. 3B, E). Fish community stability did not change through time, while population asynchrony increased during the second half of the time series (Fig. 3C, F). Trophic asynchrony between phytoplankton and zooplankton herbivores changed through time following a unimodal curve (Fig. 3G). No significant trends over time were recorded in the synchrony between Cladocera and arctic char, nor in the synchrony between Cladocera and brown trout (Fig. 3H, I).

Earlier ice-off was associated with increasing phytoplankton diversity, declining fish CPUE, non-linear changes in fish composition, diversity and stability (Table 1), and trophic asynchrony between phytoplankton biovolume and zooplankton herbivore density (Table 2). Later ice-on was associated with directional changes in zooplankton composition, non-linear changes in zooplankton population asynchrony, increased diversity and decreased stability of the fish community (Table 1), increased trophic asynchrony between phytoplankton biovolume and zooplankton herbivore and decreased trophic asynchrony between Cladocera and arctic char (Table 2). Warmer growing seasons were associated with directional changes in fish composition, declines in fish community stability (Table 1), and declines in trophic asynchrony between Cladocera and arctic char (Table 2). Larger spring floods were associated with declines in phytoplankton community stability, and non-linear changes in zooplankton density (Table 1) and trophic asynchrony between Cladocera and arctic char (Table 2).

**Table 1:** Relationships between biotic metrics and climatic variables as estimated by generalized additive models (GAMs)

	Ice off			Ice on			T growing season			Spring discharge		
	Dir.	F	*p*	Dir.	F	*p*	Dir.	F	*p*	Dir.	F	*p*
*Phytoplankton*												
Biovolume		2.30	0.179		1.24	0.295		0.44	0.515		2.42	0.089
Richness		0.40	0.533		2.27	0.145		0.00	0.997		0.53	0.472
Composition		2.28	0.108		0.02	0.883		1.29	0.268		0.01	0.951
Shannon diversity	**-**	**11.17**	**0.003**		0.06	0.854		0.10	0.752		1.03	0.319
Comm stability		0.51	0.502		0.90	0.460		6.70	0.072	-	**18.76**	**0.005**
Pop. asynchrony		0.38	0.696		2.46	0.240		2.93	0.138		0.42	0.646
*Zooplankton*												
Density		0.00	0.955		0.03	0.863		0.54	0.470	-+	**3.44**	**0.046**
Richness		0.52	0.491		2.91	0.102		0.11	0.746		0.05	0.823
Composition		1.47	0.238	**+**	**4.67**	**0.041**		2.05	0.165		2.34	0.137
Shannon diversity		0.20	0.655		1.51	0.232		1.04	0.320		2.72	0.087
Comm stability		6.71	0.251		65.23	0.110		5.63	0.305		5.40	0.325
Pop. asynchrony		2.71	0.109	-+	**5.42**	**0.043**		0.01	0.943		2.67	0.163
*Fish*												
CPUE	**+**	**9.31**	**0.001**		2.54	0.099		0.35	0.563		2.25	0.148
Composition	**+-**	**5.78**	**0.011**		1.09	0.250	**+**	**3.48**	**0.045**		0.31	0.586
Shannon diversity	**+-**	**6.72**	**0.002**	**+**	**4.47**	**0.047**		2.00	0.162		0.29	0.593
Comm stability	**+-**	**18.90**	**0.005**	-	**23.49**	**0.005**	-	**25.32**	**0.007**		0.20	0.676
Pop. asynchrony		0.90	0.489		0.00	0.954		1.26	0.305		3.86	0.097

Each biotic metric was modelled separately as a function of four abiotic variables fitted as smooth terms: timing of ice break-up (Ice off) and formation (Ice-on), mean growing-season temperature (T growing season), and spring discharge. The direction of the effect (Dir.), F-statistics, and p-value of the smooth terms are reported. Significant values are shown in bold

**Table 2:** Relationships between trophic asynchrony and climatic variables as estimated by generalized additive models (GAMs)

	Ice off			Ice on			T growing season			Spring discharge		
	Dir.	F	*p*	Dir.	F	*p*	Dir.	F	*p*	Dir.	F	*p*
Phytoplankton - herbivores	-+	**5.85**	**0.030**	+	**20.49**	**0.017**		1.05	0.371		3.25	0.132
Cladocera - Arctic char		1.59	0.297	-	**42.64**	**0.005**	-	**91.00**	**0.002**	**-+**	**97.09**	**0.002**
Cladocera - Brown trout		3.50	0.166		1.40	0.311		1.21	0.334		6.24	0.073

Each trophic asynchrony metric (phytoplankton – herbivores; Cladocera - Arctic char; Cladocera - Brown trout) was modelled separately as a function of four abiotic variables fitted as smooth terms: timing of ice break-up (Ice off) and formation (Ice-on), mean growing-season temperature (T growing season), and spring discharge. The direction of the correlation (Dir.), F-statistics, and p-value of the of the smooth terms are reported. Significant values are shown in bold

A set of biotic metrics was not correlated to any of the climatic variables (Tables 1 and 2). These metrics were phytoplankton biovolume, richness, composition and population asynchrony, zooplankton richness, diversity and community stability, fish population asynchrony, and trophic asynchrony between Cladocera and brown trout.

## Discussion

The pelagic biota of Lake Atnsjøen underwent considerable changes over the past decades, despite the lake’s remote location and minimal direct human impact in its catchment. Our results showed that both the structure and dynamics of lake biota shifted through time. Some of these changes were significantly linked with variation in climate-related environmental variables. Long-term abiotic data showed a decline in ice-cover duration, driven primarily by earlier ice-off dates, as well as increasing growing-season temperatures. However, these trends were not statistically evident within the shorter period covered by the biological analyses. Thus, the associations identified by our explanatory models should be interpreted as evidence that certain components of the biota are sensitive to interannual variation in ice phenology and climate rather than as evidence of direct responses to directional environmental change during the study period. Moreover, these associations were limited to a subset of metrics, indicating that while climate variability was consistent with parts of the observed biological change, it did not fully account for the overall temporal patterns.

### Phytoplankton

We observed an increasing trend in phytoplankton biovolume and richness over time. However, contrary to our expectation, these patterns were not correlated with the studied climatic variables. Among the structural metrics, only Shannon diversity was correlated with ice phenology (higher diversity with earlier ice-off), and among the metrics of community dynamics, only community stability was negatively correlated with the magnitude of spring flood. These results partially contrast the findings of Weyhenmeyer et al. (2013), who identified ice phenology (in that case the duration of the open-water period) as the best predictor of phytoplankton species richness and biomass. While our findings align with those of Adrian et al. (1999), who observed that overall plankton biomass is not directly linked to ice-cover duration, the discrepancy with Weyhenmeyer et al. (2013) may be due to differences in the gradient of ice cover duration. Our study examined a relatively narrow range of open-water duration (164 to 215 days), which did not reach the 220-day threshold identified by Weyhenmeyer et al. (2013) as critical for increased phytoplankton biomass in Swedish lakes. This smaller range may limit the ability to detect the nonlinear relationships observed in their study. Thus, it is possible that further reductions in ice-cover duration at Lake Atnsjøen could lead to a more pronounced increase in phytoplankton biovolume.

Ice phenology has been shown to influence the timing and magnitude of spring blooms differentially across various planktonic groups (Adrian et al. 1999). In our study, shorter ice cover duration was associated with higher Shannon diversity of phytoplankton. This pattern suggests that an extended growing season may foster a more balanced and even taxonomic distribution, enhancing overall diversity.

Greater spring discharge was associated with reduced phytoplankton community stability. Spring floods can influence lake ecosystems through multiple and potentially opposing mechanisms. Elevated discharge during snowmelt may increase nutrient inputs from the catchment, potentially stimulating primary production (Bergström 2020). On the other hand, extreme flood events, particularly those involving upstream erosion or landslides, can substantially increase turbidity (Brettum and Halvorsen 2004), reducing light availability and constraining photosynthesis. In addition, high discharge increases flow-through rates and shortens water residence time. For small and weakly motile organisms such as phytoplankton, these hydrological conditions may elevate washout losses and disrupt population persistence. Together, these processes likely increased year-to-year variability in biovolume, even if mean production did not decline, thereby lowering community stability under high spring discharge.

One striking observation from our study was the prominent increase in biovolume and incidence of cyanobacteria in Lake Atnsjøen towards the end of the timeseries. This is in line with reports that cyanobacteria are becoming an increasing challenge not only in eutrophic systems but potentially also in oligotrophic systems, including northern, nutrient poor lakes where their abundance appears to be rising (Favot et al. 2019; Freeman et al. 2020). Although the concentrations observed in Lake Atnsjøen are relatively low, some of the taxa recorded (i.e., *Anabaena*,* Phormidium*,* Tychonema bornetii*, and *Merismopedia*) are potentially toxin producing (Shams et al. 2015; Chorus and Welker 2021). While current levels are unlikely to pose immediate problems, their increasing presence may warrant attention as they could contribute to future ecological and water-quality challenges. While our study does not identify a definitive explanation for the increase in cyanobacteria in Lake Atnsjøen, the trend may be influenced by climate-related processes such as lake warming, increased water column stability, and increased frequency and intensity of storm events (Reinl et al. 2021).

### Zooplankton

Contrary to our expectation, we did not find that an increasing phytoplankton biovolume through time was reflected in an increase in total zooplankton density. It is unlikely that the lack of zooplankton response to increased phytoplankton biovolume was caused by an increased top-down control by fish, as the arctic char population, the main fish predator of zooplankton in Lake Atnsjøen (Saksgård and Hesthagen 2004), declined by approximately half over the study period. Our results instead align with the findings of Adrian et al. (1999), who observed that while shorter ice durations led to earlier abundance peaks for some zooplankton groups, the magnitude of these peaks remained unchanged. The temporal resolution of our data (five sampling dates per year that were pooled into annual samples) did not allow for a detailed investigation of shifts in peak timing. More frequent data points may have provided better insights into potential shifts in peak phenology.

Although total density of zooplankton did not show any trend, we observed a change in zooplankton community composition, with increasing densities of both cyclopoids (*Cyclops scutifer*) and calanoids (*Arctodiaptomus laticeps*), and a decline in the dominance of rotifers through time. Independently of these temporal changes, variation in zooplankton community composition was significantly associated with interannual variation in ice phenology, with shifts in community composition observed in years with later ice-on dates, indicative of shorter ice-cover duration in our study system. Under reduced planktivory pressure, an increase in fish-vulnerable taxa, particularly larger-bodied cladocerans, would be expected. However, we found no significant increases in Cladocera abundance, suggesting that altered fish predation pressure was unlikely to be the primary driver of the observed zooplankton dynamics. The observed shifts were therefore more consistent with selective responses to environmental variability than with strong top-down control. These taxon-specific trends observed in our study are consistent with the findings of Carter and Schindler (2012), who reported no clear trends in end-of-season zooplankton densities under changes in ice cover, but did observe taxon-specific responses to warming. In our study, the observed decrease in rotifer density may be explained by predation from the increasing cyclopoid population, as cyclopoids are efficient rotifer predators (Brandl 2005). Previous studies have shown that cyclopoids may benefit from delayed ice-on conditions, which can enhance winter survival and energy storage (Hebert et al. 2021), and that higher temperatures may promote their abundance despite potential negative effects of earlier spring ice-off (Carter and Schindler 2012). In contrast, calanoids seem to benefit from earlier spring ice-off (Carter and Schindler 2012). Among these, the diaptomids, which comprise most of the northern freshwater calanoids, are mainly herbivorous (Merz et al. 2023) and hence may, in our study system, have benefitted from the concurrent increase in phytoplankton biovolume.

Our trend analysis also showed that zooplankton stability declined from the beginning of the time series, particularly until around 2010. This pattern largely reflected pronounced interannual fluctuations in rotifer density during the first part of the study period.

### Fish

Fish metrics showed stronger associations with the studied environmental variables. Fish CPUE declined over time and was negatively associated with ice-cover duration, with lower CPUE observed in years with earlier ice-off. Additionally, community composition shifted over time, transitioning from a dominance of arctic char to a dominance of brown trout. Community composition was additionally associated with variation in growing-season temperature. These results align with the findings that brown trout are replacing arctic char in anadromous systems in northern Norway and Iceland (Svenning et al. 2022). Arctic char, a cold-water stenothermic species, has been observed outcompeting brown trout in cold, ultra-oligotrophic lakes (Finstad et al. 2011), where extended ice cover favours its dominance and leads to declining brown trout biomass (Helland et al. 2011). However, as a lake-spawner, arctic char eggs and alevins are particularly sensitive to temperature increases, which may lead to reduced survival and population declines and displacement from littoral habitats with warming (Elliott and Elliott 2010; Eloranta et al. 2013; Beuvard et al. 2022). Accordingly, projections indicate that more than 40% of Scandinavian populations (and possibly up to 80%) could face extirpation by the end of the 21st century due to warming (Muhlfeld et al. 2024).

Arctic char has also been shown to switch from littoral to a more pelagic trophic niche when coexisting with brown trout, a strong and predominantly benthivore competitor (Eloranta et al. 2013). The combination of warming and competition could thus have hampered arctic char populations and favoured brown trout, which are less vulnerable to warming (Elliott and Elliott 2010).

Increased diversity of the fish community was associated with later ice cover onset; moreover, diversity increased over time, reflecting increased catches of common minnow. However, the common minnow population remained sparse. As a primarily littoral species, its abundance was likely limited by the lake’s steep shoreline and restricted shallow habitat (Hesthagen and Sandlund 2004).

Our explanatory models indicated that fish community stability was lower in years with shorter ice-cover duration (i.e. later ice-on dates) and warmer conditions. In contrast, population synchrony showed a temporal trend, with populations becoming increasingly asynchronous during the second half of the time series. Although the GAM analyses did not identify environmental drivers of this temporal change in synchrony, the increasing asynchrony may nevertheless reflect differences in how the two dominant fish species respond to environmental variability (Thompson et al. 2015). As discussed above, brown trout, with their greater tolerance for warming, seem to be advantaged by the changed conditions relative to arctic char. In contrast, arctic char was at a disadvantage with these shifts, resulting in increasingly asynchronous dynamics between the two species. These patterns of community stability and population asynchrony suggest that environmental change may have reduced the stabilizing effect of compensatory dynamics (Vasseur et al. 2014), leading to decreased community stability despite increased asynchrony (Gonzalez and Loreau 2009).

### Trophic synchrony

As expected, trophic asynchrony between phytoplankton and zooplankton herbivores was greater in years with shorter ice-cover duration (i.e. earlier ice-off). In ice-covered lakes like Atnsjøen, warming is predicted to amplify this asynchrony, as phytoplankton blooms are primarily driven by light availability after ice-off, and zooplankton consumer peaks are primarily regulated by water temperature, two processes that respond to warming at different rates (Gronchi et al. 2023). Although our data lack the temporal resolution to analyse the timing of these peaks (i.e. intra-year seasonal patterns), the observed increase in asynchrony of yearly averaged abundances in years with shorter ice-cover duration suggests that warming may have disrupted the trophic synchrony between phyto- and zooplankton. The different rates at which phenological change may manifest among trophic levels can lead to temporal mismatch in trophic interactions (Thackeray et al. 2010). For example, decoupling between the timing of diatom blooms and peak *Daphnia* abundance has been documented in Lake Washington (USA; Winder and Schindler 2004). Such mismatches may potentially reduce the efficiency of energy transfer in the food-chain.

The patterns of asynchrony between the populations of the two dominant fish species and their zooplankton prey seem to be the result of warming and competition. As discussed above, warming and competition with brown trout likely caused a shift in the trophic niche of arctic char, making it more reliant on zooplankton (Cladocera) (Eloranta et al. 2013; Beuvard et al. 2022). Indeed, the arctic char population was more synchronous with Cladocera under shorter ice cover and warmer temperatures. Meanwhile, brown trout, being a stronger benthivore competitor under warming, is likely to feed more on benthic invertebrates, which may have resulted in its population being less dependent on and synchronous with Cladocera.

## Conclusions

Our study demonstrates that lake pelagic biodiversity and trophic coupling have undergone considerable changes over the past three decades, even in an oligotrophic lake with minimal direct human impacts in its catchment. The observational nature of this study, along with the focus on a single lake, limits our ability to infer causal relationships or fully resolve the driving forces behind these changes. Although only a subset of biological metrics was significantly associated with climate-related variables, including ice phenology, temperature, and hydrological conditions, these associations indicate that some components of the biota were sensitive to interannual abiotic variability. Long-term abiotic records further showed declining ice-cover duration and increasing growing-season temperatures, suggesting that these environmental sensitivities may be relevant under ongoing environmental change. At the same time, not all temporal trends were linked to the examined environmental drivers, indicating that additional processes likely contributed to the observed changes. While we cannot rule out the influence of other non-climate drivers, such as changes in nutrient loads, the lake’s remote location and minimal direct human impact suggest that direct anthropogenic influences are unlikely to have been the main drivers of change. Instead, it is possible that other climate-related processes within the catchment, such as mountain greening (i.e., increases in vegetation cover and primary productivity in alpine and subalpine areas), may also have played a role in shaping the observed biotic changes. In conclusion, our results indicate significant restructuring of pelagic communities under ongoing climate change and highlight the need for long-term monitoring to track those changes and anticipate future shifts.

## Supplementary Information

Below is the link to the electronic supplementary material.

Supplementary Material 1

## Data Availability

Ice-cover data were obtained from http://www.iskart.no, daily air temperatures were downloaded from https://api.nve.no/doc/gridtimeseries-data-gts, and daily lake outlet discharges from https://sildre.nve.no. The phytoplankton data used in this work are publicly available at https://vannmiljo.miljodirektoratet.no (waterLocationID = 38005). The zooplankton and fish data that were used in this study are available at https://osf.io/ydwgv.

## Code Availability

All R functions and libraries are cited in the text. Custom code is available at https://osf.io/ydwgv.

## References

[CR1] Adrian R, Walz N, Hintze T et al (1999) Effects of ice duration on plankton succession during spring in a shallow polymictic lake. Freshw Biol 41:621–634. 10.1046/j.1365-2427.1999.00411.x

[CR2] Bergström A-K (2020) Hydrological controls on pelagic food structure—From shunts to chemostats as caused by runoff magnitudes and frequency of episodes. Hydrol Process 34:4150–4155. 10.1002/hyp.13886

[CR3] Beuvard C, Imsland AKD, Thorarensen H (2022) The effect of temperature on growth performance and aerobic metabolic scope in Arctic charr, *Salvelinus alpinus* (L). J Therm Biol 104:103117. 10.1016/j.jtherbio.2021.103117 35180951

[CR4] Brænd B (1989) Sollia. 1. Eldre generell historie, garder og slekter i Øverdalen skolekrets [Sollia 1. Older general history, farms and families in Øverdalen school district]. Stor-Elvdal kommune

[CR5] Brænd B (2007) Sollia. 2. Generell historie 1862–1945, garder i statsallmenningen og slekter fra Setningssjøen til Holtet i Atnbrua skolekrets [Sollia 2. General history 1862–1945, farms in the state community and families from Lake Setningssjøen to Holtet in Atnbrua school district]. Stor-Elvdal kommune

[CR6] Brænd B (2009) Sollia 3. Generell historie etter 1945, garder og slekter i Atnelien i Atnbrua skolekrets [Sollia 3. General history after 1945, farms and families in Atnelien in Atnbrua school district]. Stor-Elvdal kommune

[CR7] Brandl Z (2005) Freshwater Copepods and Rotifers: Predators and their Prey. Hydrobiologia 546:475–489. 10.1007/s10750-005-4290-3

[CR8] Brettum P, Halvorsen G (2004) The phytoplankton of Lake Atnsjøen, Norway – a long-term investigation. Hydrobiologia 521:141–147. 10.1023/B:HYDR.0000026356.09421.e3

[CR9] Caldwell TJ, Chandra S, Feher K et al (2020) Ecosystem response to earlier ice break-up date: Climate-driven changes to water temperature, lake-habitat-specific production, and trout habitat and resource use. Glob Change Biol 26:5475–5491. 10.1111/gcb.15258 32602183

[CR10] Carter JL, Schindler DE (2012) Responses of Zooplankton Populations to Four Decades of Climate Warming in Lakes of Southwestern Alaska. Ecosystems 15:1010–1026. 10.1007/s10021-012-9560-0

[CR11] Chorus I, Welker M (eds) (2021) Toxic Cyanobacteria in Water: A Guide to Their Public Health Consequences, Monitoring and Management, 2nd edn. CRC, London

[CR12] De Senerpont Domis LN, Elser JJ, Gsell AS et al (2013) Plankton dynamics under different climatic conditions in space and time. Freshw Biol 58:463–482. 10.1111/fwb.12053

[CR13] Elliott JM, Elliott JA (2010) Temperature requirements of Atlantic salmon Salmo salar, brown trout Salmo trutta and Arctic charr Salvelinus alpinus: predicting the effects of climate change. J Fish Biol 77:1793–1817. 10.1111/j.1095-8649.2010.02762.x 21078091

[CR14] Eloranta AP, Knudsen R, Amundsen P-A (2013) Niche segregation of coexisting Arctic charr (*Salvelinus alpinus*) and brown trout (*Salmo trutta*) constrains food web coupling in subarctic lakes. Freshw Biol 58:207–221. 10.1111/fwb.12052

[CR15] Favot EJ, Rühland KM, DeSellas AM et al (2019) Climate variability promotes unprecedented cyanobacterial blooms in a remote, oligotrophic Ontario lake: evidence from paleolimnology. J Paleolimnol 62:31–52. 10.1007/s10933-019-00074-4

[CR16] Finstad AG, Forseth T, Jonsson B et al (2011) Competitive exclusion along climate gradients: energy efficiency influences the distribution of two salmonid fishes. Glob Change Biol 17:1703–1711. 10.1111/j.1365-2486.2010.02335.x

[CR100] Freeman EC, Creed IF, Jones B, Bergström AK (2020) Global changes may be promoting a rise in select cyanobacteria in nutrient-poor northern lakes. Glob Change Biol 26:4966–4987. 10.1111/gcb.15189 32445590

[CR17] Gonzalez A, Loreau M (2009) The Causes and Consequences of Compensatory Dynamics in Ecological Communities. Annu Rev Ecol Evol Syst 40:393. 10.1146/annurev.ecolsys.39.110707.173349

[CR18] Gronchi E, Straile D, Diehl S et al (2023) Impact of climate warming on phenological asynchrony of plankton dynamics across Europe. Ecol Lett 26:717–728. 10.1111/ele.14190 36870064

[CR19] Halvorsen G (2004) Some physical and chemical characteristics of Lake Atnsjøen. Hydrobiologia 521:129–140. 10.1023/B:HYDR.0000026355.60067.6b

[CR20] Halvorsen G, Dervo BK, Papinska K (2004) Zooplankton in Lake Atnsjøen 1985–1997. Hydrobiologia 521:149–175

[CR21] Hampton SE, Galloway AWE, Powers SM et al (2017) Ecology under lake ice. Ecol Lett 20:98–111. 10.1111/ele.12699 27889953

[CR22] Hebert M-P, Beisner BE, Rautio M, Fussmann GF (2021) Warming winters in lakes: Later ice onset promotes consumer overwintering and shapes springtime planktonic food webs. Proc Natl Acad Sci (PNAS) 118:e2114840118. 10.1073/pnas.2114840118 34810251 PMC8694056

[CR23] Helland IP, Finstad AG, Forseth T et al (2011) Ice-cover effects on competitive interactions between two fish species: Ice-cover and competition. J Anim Ecol 80:539–547. 10.1111/j.1365-2656.2010.01793.x 21198589

[CR24] Hesthagen T, Sandlund OT (2004) Fish distribution in a mountain area in south-eastern Norway: human introductions overrule natural immigration. Hydrobiologia 521:49–59. 10.1023/B:HYDR.0000026350.93171.ba

[CR25] IPCC (2019) IPCC Special Report on the Ocean and Cryosphere in a Changing Climate. IPCC, Cambridge University Press, Cambridge, UK and New York, NY, USA

[CR26] Ives AR, Carpenter SR (2007) Stability and Diversity of Ecosystems. Science 317:58–62. 10.1126/science.1133258 17615333

[CR27] Jensen TC, Zawiska I, Oksman M et al (2020) Historical human impact on productivity and biodiversity in a subalpine oligotrophic lake in Scandinavia. J Paleolimnol 63:1–20. 10.1007/s10933-019-00100-5

[CR28] Jeppesen E, Madsen EA, Jensen JP, Anderson N (1996) Reconstructing the past density of planktivorous fish and trophic structure from sedimentary zooplankton fossils: a surface sediment calibration data set from shallow lakes. Freshw Biol 36:115–127. 10.1046/j.1365-2427.1996.00085.x

[CR29] L’Abée-Lund JH, Vøllestad LA, Brittain JE et al (2021) Geographic variation and temporal trends in ice phenology in Norwegian lakes during the period 1890–2020. Cryosphere 15:2333–2356. 10.5194/tc-15-2333-2021

[CR30] Larsen S, Joyce F, Vaughan IP et al (2024) Climatic effects on the synchrony and stability of temperate headwater invertebrates over four decades. Glob Change Biol 30:e17017. 10.1111/gcb.17017 37933478

[CR31] Legendre P, Gallagher ED (2001) Ecologically meaningful transformations for ordination of species data. Oecologia 129:271–280. 10.1007/s004420100716 28547606

[CR32] Merz E, Saberski E, Gilarranz LJ et al (2023) Disruption of ecological networks in lakes by climate change and nutrient fluctuations. Nat Clim Chang 1–8. 10.1038/s41558-023-01615-6 PMC10079529 37038592

[CR33] Muhlfeld CC, Cline TJ, Finstad AG et al (2024) Climate change vulnerability of Arctic char across Scandinavia. Glob Change Biol 30:e17387. 10.1111/gcb.17387 38971982

[CR34] O’Connor RF, McMeans BC, Rooney N et al (2023) Species portfolio effects dominate seasonal zooplankton stabilization within a large temperate lake. Ecology 104:e3889. 10.1002/ecy.3889 36208063

[CR35] O’Reilly CM, Sharma S, Gray DK et al (2015) Rapid and highly variable warming of lake surface waters around the globe. Geophys Res Lett 42 :10,773 – 10,781. 10.1002/2015GL066235

[CR36] Paerl HW, Huisman J (2008) Blooms Like It Hot. Science 320:57–58. 10.1126/science.1155398 18388279

[CR37] R Core Team (2019) R: A language and environment for statistical computing. R Foundation for Statistical Computing, Vienna, Austria. https://www.R-project.org/

[CR38] Rao X, Chen J, Wang S et al (2024) Population asynchrony within and between trophic levels have contrasting effects on consumer community stability in a subtropical lake. J Anim Ecol 93:1593–1605. 10.1111/1365-2656.14176 39268554

[CR39] Reinl KL, Brookes JD, Carey CC et al (2021) Cyanobacterial blooms in oligotrophic lakes: Shifting the high-nutrient paradigm. Freshw Biol 66:1846–1859. 10.1111/fwb.13791

[CR40] Saksgård R, Hesthagen T (2004) A 14-year study of habitat use and diet of brown trout (Salmo trutta) and Arctic charr (Salvelinus alpinus) in Lake Atnsjøen, a subalpine Norwegian lake. Hydrobiologia 521:187–199

[CR41] Shams S, Capelli C, Cerasino L et al (2015) Anatoxin-a producing *Tychonema* (Cyanobacteria) in European waterbodies. Water Res 69:68–79. 10.1016/j.watres.2014.11.006 25437339

[CR42] Simpson GL, Singmann H (2022) gratia: Graceful ’ggplot’-Based Graphics and Other Functions for GAMs Fitted Using mgcv. R package version 0.9.0. https://gavinsimpson.github.io/gratia/

[CR43] Skålevåg A, Vormoor K (2021) Daily streamflow trends in Western versus Eastern Norway and their attribution to hydro-meteorological drivers. Hydrol Process 35:e14329. 10.1002/hyp.14329

[CR45] Svenning M-A, Falkegård M, Dempson JB et al (2022) Temporal changes in the relative abundance of anadromous Arctic charr, brown trout, and Atlantic salmon in northern Europe: Do they reflect changing climates? Freshw Biol 67:64–77. 10.1111/fwb.13693

[CR44] Svenning MA, Bjørvik ET, Godiksen JA et al (2024) Expected Climate Change in the High Arctic—Good or Bad for. Arct Charr? Fishes 9:8. 10.3390/fishes9010008

[CR46] Thackeray SJ, Sparks TH, Frederiksen M et al (2010) Trophic level asynchrony in rates of phenological change for marine, freshwater and terrestrial environments. Glob Change Biol 16:3304–3313. 10.1111/j.1365-2486.2010.02165.x

[CR47] Thibaut LM, Connolly SR (2013) Understanding diversity–stability relationships: towards a unified model of portfolio effects. Ecol Lett 16:140–150. 10.1111/ele.12019 23095077 PMC3588152

[CR48] Thompson PL, Beisner BE, Gonzalez A (2015) Warming induces synchrony and destabilizes experimental pond zooplankton metacommunities. Oikos 124:1171–1180. 10.1111/oik.01945

[CR49] Tilman D (1999) The Ecological Consequences of Changes in Biodiversity: A Search for General Principles. Ecology 80:1455–1474. 10.1890/0012-9658(1999)080[1455:TECOCI]2.0.CO;2

[CR50] Tvede AM, Halvorsen G (2004) Introduction to the Atna research area. Hydrobiologia 521:1–4. 10.1023/B:HYDR.0000026346.95232.98

[CR51] Vasseur DA, Fox JW, Gonzalez A et al (2014) Synchronous dynamics of zooplankton competitors prevail in temperate lake ecosystems. Proceedings of the Royal Society B: Biological Sciences 281:20140633. 10.1098/rspb.2014.0633 PMC4083788 24966312

[CR52] Wang W, Lee X, Xiao W et al (2018) Global lake evaporation accelerated by changes in surface energy allocation in a warmer climate. Nat Geosci 11:410–414. 10.1038/s41561-018-0114-8

[CR53] Weyhenmeyer GA (2009) Do warmer winters change variability patterns of physical and chemical lake conditions in Sweden? Aquat Ecol 43:653–659. 10.1007/s10452-009-9284-1

[CR54] Weyhenmeyer GA, Peter H, Willén E (2013) Shifts in phytoplankton species richness and biomass along a latitudinal gradient – consequences for relationships between biodiversity and ecosystem functioning. Freshw Biol 58:612–623. 10.1111/j.1365-2427.2012.02779.x

[CR55] Winder M, Schindler DE (2004) Climate Change Uncouples Trophic Interactions in an Aquatic Ecosystem. Ecology 85:2100–2106

[CR56] Wood SN (2011) Fast stable restricted maximum likelihood and marginal likelihood estimation of semiparametric generalized linear models. J Royal Stat Society: Ser B (Statistical Methodology) 73:3–36. 10.1111/j.1467-9868.2010.00749.x

[CR57] Woolway RI, Kraemer BM, Lenters JD et al (2020) Global lake responses to climate change. Nat Rev Earth Environ 1:388–403. 10.1038/s43017-020-0067-5

