# Supplementary material for: Impacts of climate and reduced ice cover on trophic structure and community dynamics in a high latitude lake
Source: Oecologia. 2026 Aug 14;208(9):116. doi: 10.1007/s00442-026-05957-8 (PMC13476355; doi:10.1007/s00442-026-05957-8)
Supplement: Supplementary file 1 [file 442_2026_5957_MOESM1_ESM.docx]

**Impacts of climate and reduced ice cover on trophic structure and community dynamics in a high latitude lake**

Francesca Pilotto ^1*^, Knut Marius Myrvold ^2^, Randi Saksgård ^3^, Knut Andreas Eikland ^2^, Birger Skjelbred ^4^, Stefano Larsen ^5-6^, Gunnar Halvorsen, Trygve Hesthagen, Thomas C. Jensen ^1^

^1^ Norwegian Institute for Nature Research (NINA). Oslo, Norway

^2^ Norwegian Institute for Nature Research (NINA). Lillehammer, Norway

^3^ Norwegian Institute for Nature Research (NINA). Trondheim, Norway

^4^ Norwegian Institute for Water Research (NIVA). Oslo, Norway

^5^ Research and Innovation Centre – Edmund Mach Foundation. San Michele all'Adige, Italy

^6^ NBFC- National Biodiversity Future Center. Palermo, Italy

* Corresponding author.

**Supplementary materials**

**Supplementary Figure 1.** Map of Lake Atnsjøen with survey locations. The star shows the sampling station for phytoplankton and zooplankton. The labels show the locations of the pelagic (P1-3) and benthic (B1-8) nets for test fishing.

**Supplementary Figure 2.** CPUE of fish populations in the benthic (red) and pelagic (blue) nets and their average values (black).

**Supplementary Figure 3.** Principal Coordinates Analysis (PCoA) for phytoplankton (A–B), zooplankton (C–D) and fish (E–F) communities. The proportion of variance explained by each axis is indicated in the panels. For clarity, only zooplankton taxa at the extremes of the ordination space are labelled in panel C. Zooplankton labels: P.rema = *Polyarthra remata*; P.doli = *Polyarthra dolichoptera*; Poly.pedi = *Polyphemus pediculus*; Brac.sp = *Brachionus sp.*; Arct.lati = *Arctodiaptomus laticeps*; Filin.sp = *Filinia sp*.; Cycl.scut = *Cyclops scutifer*; Noth.sp = *Notholca sp*.; Kera.coch = *Keratella cochlearis*; Trich.sp = *Trichocerca sp*; Ceph.sp = *Cephalodella sp*.; Asco.sp = *Ascomorpha sp*.; Acan.vern = *Acanthocyclops vernalis*; Rota.sp = *Rotaria sp*.; Sync.pect = *Synchaeta pectinata*; Euch.sp = *Euchlanis sp*.; Chyd.sp = *Chydorus sp*.; S.obo = *Synchaeta oblonga*; K.serr = *Keratella serrulata*.

**Supplementary Figure 4.** Changes in abiotic variables through time. The full time series of abiotic variables are shown, with the period corresponding to the biological data (1994–2022) shaded in yellow. Points show observed values; blue lines and ribbons show the fitted GAM smooth and 95% C.I., only significant smooths are shown (p < 0.05). When the analysis was restricted to 1994–2022, only ice-cover duration showed a significant trend. Note that for this figure, growing-season temperature was defined as the mean temperature from ice-off to 30 September, rather than from ice-off to the September sampling date as in the main analyses, because this definition allowed the full climatic time series to be analysed.

**Supplementary Figure 5.** Changes in the biovolume (mm^3^/m^3^) of phytoplankton biotic groups (A-J), changes in the densities (ind./l) of zooplankton groups (K-N; nauplii were excluded) and changes in CPUE (ind.) of the two fish species (O-R). Points show observed values; blue lines and ribbons show the fitted GAM smooth and 95% C.I., only significant smooths are shown.

**Supplementary Figure 6.** Occurrence of cyanobacteria throughout the study period. Vertical lines indicate the five sampling occasions each year, and red dots represent the biovolume of cyanobacteria when present.
